# Plant-Mediated Synthesis of Calcium Oxide Nanoparticles Using Syzygium cumini Fruit Extract and Assessment of Anti-inflammatory Activity: An In Vitro Study

**DOI:** 10.7759/cureus.112308

**Published:** 2026-07-08

**Authors:** Srividhya Srinivasan, Saraswathi K, Rajeshkumar Shanmugam, Anand Bala, Thirumal Kumar D

**Affiliations:** 1 Oral Medicine and Radiology, Meenakshi Ammal Dental College and Hospital, Chennai, IND; 2 Oral and Maxillofacial Radiology, Meenakshi Ammal Dental College and Hospital, Chennai, IND; 3 Nanobiomedicine Lab, Centre for Global Health Research, Saveetha Medical College and Hospital, Saveetha Institute of Medical and Technical Sciences, Chennai, IND; 4 Oral Medicine and Radiology, Ragas Dental College, Chennai, IND; 5 Research and Development, Meenakshi Academy of Higher Education and Research, Chennai, IND

**Keywords:** anti-inflammatory activity, bovine denaturation, calcium oxide nanoparticles, green synthesis, membrane stabilization, protein denaturation assay, syzygium cumini

## Abstract

Background

Plant-mediated synthesis of nanoparticles has gained increasing attention as an environmentally friendly approach for developing biologically active nanomaterials. *Syzygium cumini* (Jamun) fruit is rich in polyphenols, flavonoids, and anthocyanins known for their antioxidant and anti-inflammatory properties. However, the anti-inflammatory potential of calcium oxide nanoparticles (CaO-NPs) synthesized using *S. cumini* fruit extract has not been extensively explored. The present study aimed to synthesize CaO-NPs using *S. cumini* fruit extract through a green synthesis approach and to evaluate the in vitro anti-inflammatory activity.

Methodology

CaO-NPs were synthesized using aqueous fruit extract of *S. cumini*. The anti-inflammatory activity of the extract and CaO-NPs was evaluated using the egg albumin denaturation assay, bovine serum albumin denaturation assay, and membrane stabilization assay using Diclofenac sodium as the reference standard. The percentage inhibition of protein denaturation and membrane lysis was determined spectrophotometrically.

Results

Both the fruit extract and CaO-NPs demonstrated concentration-dependent anti-inflammatory activity in all assays. At higher concentrations, CaO-NPs showed greater inhibition of protein denaturation and improved membrane stabilization compared with the fruit extract. The observed inhibition values for CaO-NPs approached those of the standard drug, indicating enhanced biological activity following nanoparticle synthesis.

Conclusions

The findings suggest that green-synthesized CaO-NPs derived from *S. cumini* fruit possess significant anti-inflammatory activity. While in vitro protein denaturation and membrane stabilization assays are well-established screening models for anti-inflammatory activity, further validation using cellular and in vivo models is essential to elucidate the underlying mechanisms and confirm the therapeutic potential of plant-mediated CaO-NPs.

## Introduction

Inflammation is a complex biological response characterized by protein denaturation, membrane instability, oxidative stress, and the release of pro-inflammatory mediators. In the oral cavity, inflammatory conditions such as oral mucositis, aphthous ulcers, and periodontal disease are associated with epithelial dammarane, lysosomal membrane disruption, and excessive cytokine production. Although non-steroidal anti-inflammatory drugs remain effective in controlling inflammation, their prolonged use is frequently associated with adverse effects, prompting the search for safer and more biocompatible therapeutic alternatives [1].

Plant-derived bioactive compounds have attracted considerable attention because of their antioxidant, anti-inflammatory, and membrane-stabilizing properties. *Syzygium cumini* (Jamun) fruit is a medicinal fruit rich in anthocyanins, flavonoids, and phenolic compounds, all of which have demonstrated significant anti-inflammatory and antioxidant properties [2]. Crude plant extracts, however, frequently have variable pharmacodynamic profiles, poor stability, low bioavailability, and quick breakdown under physiological circumstances. Their translational potential in clinical applications is limited by these constraints. A method to improve phytochemical stability and biological efficacy is the green production of nanoparticles using plant extracts. By combining the inherent reactivity of nanomaterials with phytochemical surface capping, plant-mediated metal and metal oxide nanoparticles may enhance their anti-inflammatory and antioxidant properties [3]. Green-synthesized metal and metal oxide nanoparticles have attracted considerable interest because of their ability to modulate inflammatory processes through multiple mechanisms. Silver (Ag-NPs), zinc oxide (ZnO-NPs), and copper oxide (CuO-NPs) nanoparticles synthesized using plant extracts have consistently demonstrated inhibition of heat-induced protein denaturation and stabilization of erythrocyte membranes, both of which are accepted in vitro indicators of anti-inflammatory activity [4,5]. According to research by Shanmugam et al., phytochemical-assisted metal oxide nanoparticles show improved anti-inflammatory profiles when compared to crude extracts, which lends credence to the theory that incorporating nanoparticles could increase bioactivity [6].

The majority of research on anti-inflammatory nanoparticles concentrates on Ag-NPs, ZnO-NPs, or CuO-NPs. Despite these advances, limited information is available regarding the anti-inflammatory potential of calcium oxide nanoparticles (CaO-NPs) made from fruit extracts. Although CaO-NPs have been investigated for dental and antibacterial applications, nothing is known about their ability to reduce inflammation [7]. This is a major untapped possibility because of the mineral relevance and biocompatibility of calcium-based technologies in oral tissues.

Damage to the epithelial membrane, oxidative stress, and heightened vulnerability to microbial colonization are all components of inflammatory oral diseases. Fruit-mediated CaO-NPs intended for oral anti-inflammatory uses have not been thoroughly studied, despite the exploration of nanomedicine techniques for oral mucositis [8].

Therefore, the present study aimed to synthesize CaO-NPs using *S. cumini* fruit extract through a green synthesis approach, characterize the synthesized nanoparticles, and comparatively evaluate the in vitro anti-inflammatory activity of the fruit extract and CaO-NPs using egg albumin (EA) denaturation, bovine serum albumin (BSA) denaturation, and membrane stabilization assays (MSAs).

## Materials and methods

This in vitro study was conducted between June and September 2025 in Chennai using fresh, ripe *S. cumini* fruits. The fruits were washed thoroughly, and the seeds were removed. Approximately 5 g of the deseeded fruit pulp was weighed and manually ground using a mortar and pestle to obtain a uniform, semi-solid paste. The crushed pulp was then filtered through a muslin cloth to remove fibrous material, and the resulting filtrate was made up to 50 mL with distilled water to obtain the aqueous fruit extract. The ground fruit pulp was filtered using muslin cloth, and the collected extract was made up to 50 mL using distilled water. The fruit extract was boiled for 10 minutes at 30℃. The boiled extract was used to prepare the nanoparticles.

Preparation of *Syzygium cumini* calcium oxide nanoparticles

For the study, 100 mM of calcium hydroxide solution was prepared in 50 mL distilled water and mixed with 50 mL of *S. cumini* fruit extract. The reaction mixture was maintained under continuous agitation in an orbital shaker at 450 rpm, and the color change was observed. The solution was subjected to ultraviolet (UV)-visible spectroscopy to confirm the synthesized nanoparticles. The nanoparticle solution was centrifuged for 10 minutes at 8,000 rpm after 56 hours. The pellet was collected for further research studies.

Characterization of synthesized *Syzygium cumini* calcium oxide nanoparticles

**Figure 1:**
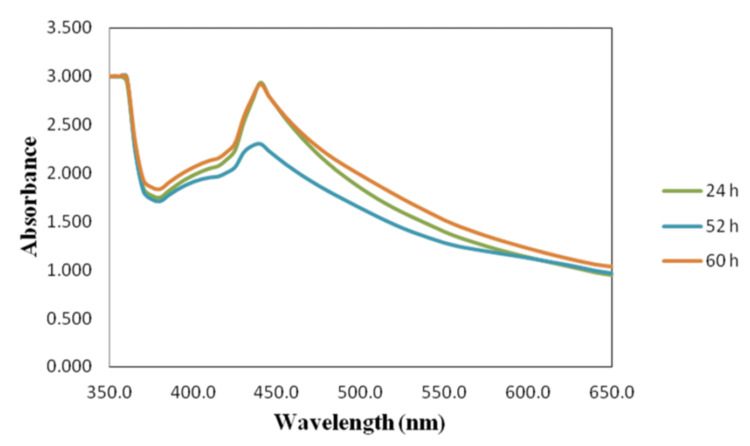
Ultraviolet-visible absorption spectra of green-synthesized calcium oxide nanoparticles synthesized using Syzygium cumini fruit extract at different incubation periods (24, 52, and 60 hours).

The biosynthesized CaO-NPs were characterized using a UV-visible spectrophotometer (UV-2600, Shimadzu Corporation, Kyoto, Japan). The absorption spectrum was recorded over a wavelength range of 200-650 nm at room temperature using a 1 cm path length quartz cuvette. The spectrum was acquired at a scan speed of 400 nm/minute with distilled water serving as the blank to confirm the appearance of characteristic absorption peaks associated with nanoscale calcium oxide (Figure 1) [9,10].

To identify the phytochemical groups involved in reducing and stabilizing the nanoparticles, Fourier transform infrared spectroscopy was performed on the dried CaO-NP sample. Crystallographic analysis of the synthesized material was performed using X-ray diffraction, enabling confirmation of the crystalline phases and lattice structure of the CaO-NPs. The external morphology, size distribution, and surface architecture of the particles were examined by scanning electron microscopy. In addition, energy-dispersive X-ray analysis was employed to verify the elemental constituents present in the nanoparticles and to confirm the successful incorporation of calcium and oxygen in the final product.

## Results

Anti-inflammatory activity: bovine serum albumin denaturation assay

*S. cumini* fruit-based CaO-NPs were assessed for their anti-inflammatory activity using the bovine serum albumin (BSA) denaturation assay. For this, 0.45 mL of BSA was mixed with 0.05 mL of multiple concentrations (10, 20, 30, 40, 50 µg/mL) of S. cumini-mediated CaO-NPs. The pH was adjusted to 6.3, which was then kept at room temperature for 10 minutes. This was followed by incubation in a water bath at 55°C for approximately 30 minutes. The standard drug used was diclofenac sodium, while dimethyl sulfoxide was used as the control. Then, the samples were measured spectrophotometrically at 660nm.

The percentage of protein denaturation was determined using the following equation: percentage inhibition = (absorbance of control - absorbance of sample)/absorbance of control × 100.

**Figure 2:**
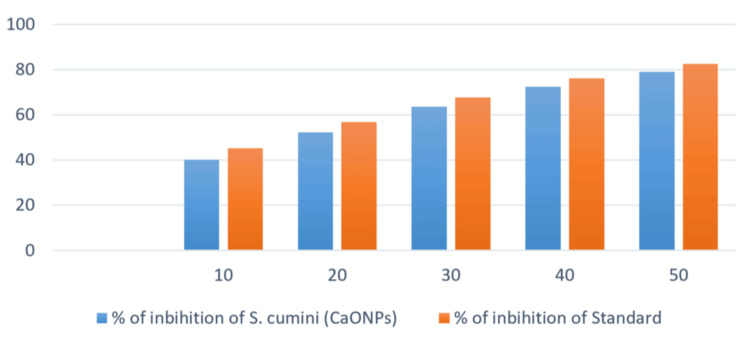
Graphical representation of the anti-inflammatory activity of Syzygium cumini calcium oxide nanoparticles using the bovine serum albumin denaturation assay.

At 10 µg/mL, inhibition was 40.12%, which increased progressively to 78.96% at 50 µg/mL. The standard drug (diclofenac sodium) exhibited slightly higher inhibition, ranging from 45.23% at 10 µg/mL to 82.54% at 50 µg/mL. At 50 µg/mL, CaO-NPs achieved 78.96% inhibition, closely approaching the standard drug (82.54%), suggesting a strong anti-inflammatory activity in vitro. The anti-inflammatory activity observed may be attributed to: (1) stabilization of protein structure by phytochemical-capped nanoparticles; (2) inhibition of heat-induced denaturation; and (3) possible antioxidant contribution of polyphenols present in *S. cumini* (Table 1, Figure 2).

**Table 1:** Bovine serum albumin denaturation assay. BSA inhibition expressed in percentage across different concentrations expressed in µg/mL for the *Syzygium cumini* CaO-NPs and the standard. *S. cumini* CaO-NPs have a comparable percentage of inhibition to that of the standard at different concentrations. BSA = bovine serum albumin; CaO-NP = calcium oxide nanoparticle

Concentrations (µg/mL)	10	20	30	40	50
BSA values of *Syzygium cumini* (CaONPs) (%)	40.12	52.34	63.58	72.45	78.96
BSA values of the standard (%)	45.23	56.78	67.83	76.14	82.54

Anti-inflammatory activity: egg albumin denaturation assay

The egg albumin (EA) denaturation assay was performed to assess the anti-inflammatory activity. For this, 0.2 mL of fresh EA was mixed with 2.8 mL of 1× phosphate buffer. Different concentrations (10, 20, 30, 40, 50 µg/mL) of *S. cumini*-mediated CaO-NPs were added to the reaction mixture. The pH was adjusted to 6.3, which was kept at room temperature for 10 minutes. This was followed by incubation in a water bath at 55°C for approximately 30 minutes. Diclofenac sodium was used as the standard group, while dimethyl sulfoxide was used as the control. Then, the samples were measured spectrophotometrically at 660 nm. The percentage of protein denaturation was calculated utilizing the same formula mentioned above.

**Figure 3:**
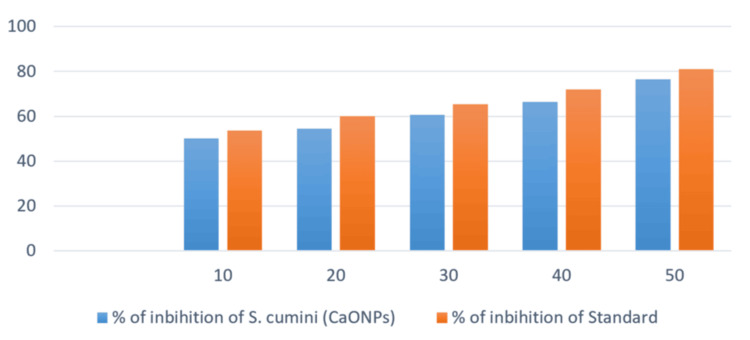
Graphical representation of the anti-inflammatory activity of S. cumini CaO-NPs using the egg albumin denaturation assay. CaO-NP = calcium oxide nanoparticle

At 10 µg/mL, inhibition was 50.17%, which increased progressively to 76.49% at 50 µg/mL. The standard drug (diclofenac sodium) exhibited slightly higher inhibition, ranging from 53.65% at 10 µg/mL to 80.99% at 50 µg/mL, which is within a close range of the standard drug (82.54%), suggesting a strong anti-inflammatory activity in vitro (Table 2, Figure 3).

**Table 2:** EA denaturation assay. Percentage inhibition observed in the EA for *Syzygium cumini*-mediated CaO-NPs and the standard drug at various concentrations (10-50 µg/mL). The synthesized *S. cumini*-mediated CaO-NPs demonstrated concentration-dependent EA denaturation activity, with percentage inhibition values comparable to those of the standard drug across the tested concentrations. EA = egg albumin; CaO-NP = calcium oxide nanoparticle

Concentrations (µg/mL)	10	20	30	40	50
EA values of *Syzygium cumini* (CaONPs) (%)	50.17	54.56	60.59	66.34	76.49
EA values of the standard (%)	53.65	59.98	65.33	71.98	80.99

Anti-inflammatory activity: membrane stabilization assay

**Figure 4:**
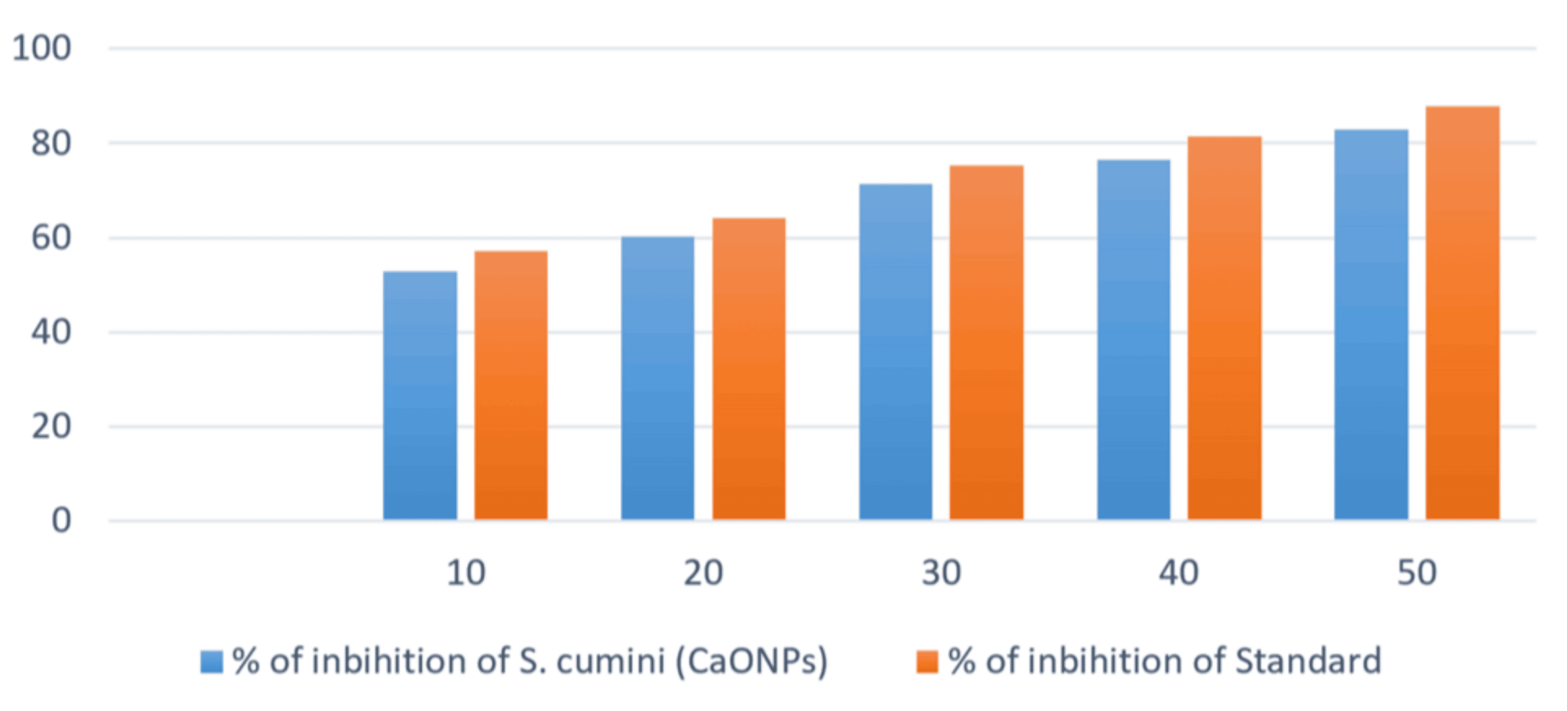
Graphical representation of the anti-inflammatory activity of Syzygium cumini CaO-NPs using the membrane stabilization assay. CaO-NP = calcium oxide nanoparticle

Membrane stabilization assay (MSA) is a commonly performed technique for assessing the membrane-stabilizing properties of natural and synthetic compounds. This assay measures the ability of a compound to stabilize the cell membrane by preventing its disruption and subsequent release of intracellular contents. The materials include human red blood cells, phosphate-buffered saline, and Tris-HCl buffer (50 mM, pH 7.4). Different concentrations of *S. cumin*-mediated CaO-NPs (10, 20, 30, 40, 50 µg/mL) were studied. CaO-NPs demonstrated significant membrane stabilization activity in a concentration-dependent manner, as inhibition increased from 52.89% at 10 µg/mL to 82.97% at 50 µg/mL, which is consistent with that of the standard, suggesting strong anti-inflammatory potential of *S. cumini*-derived CaO-NPs (Table 3, Figure 4).

**Table 3:** MSA percentage inhibition. Percentage inhibition in the MSA exhibited by *Syzygium cumini*-mediated CaO-NPs and the standard drug at various concentrations (µg/mL). *S.cumini*-mediated CaO-NPs showed anti-inflammatory activity comparable to the standard across the tested concentration range. MSA = membrane stabilization assay; CaO-NP = calcium oxide nanoparticle

Concentrations (µg/mL)	10	20	30	40	50
MSA values of *Syzygium cumini* (CaO-NPs) (%)	52.89	60.27	71.36	76.51	82.97
MSA values of the standard (%)	57.11	64.26	75.32	81.54	87.83

## Discussion

The findings of the current study demonstrate that the *S. cumini*-mediated CaO-NPs possess significant, concentration-dependent anti-inflammatory activity. The nanoparticles exhibited substantial inhibition of protein denaturation in both BSA and EA assays, as well as marked membrane stabilization activity. In all assays, the inhibitory effects increased progressively with concentration and approached the activity of the standard drug diclofenac sodium. These in vitro assays determine the following inflammatory mechanisms: membrane disruption and protein structural destabilization. The extract and nanoparticle formulation both successfully reduced inflammatory surrogate indicators, as seen by the steady rise in percentage inhibition over 10-50 µg/mL doses.

Protein denaturation is considered a major contributor to inflammatory processes because denatured proteins trigger autoantigen formation and stimulate inflammatory cascades. Therefore, inhibition of protein denaturation serves as a reliable preliminary indicator of anti-inflammatory potential. The significant inhibition observed in the present study suggests that the synthesized nanoparticles may effectively protect proteins from heat-induced structural alterations. Several studies have reported similar concentration-dependent inhibition profiles for plant-mediated metal and metal oxide nanoparticles. In EA and BSA tests, Varghese et al. showed that Ag-NPs and ZnO-NPs made from *Ocimum* species considerably reduced protein denaturation and membrane lysis, with activity comparable to that of diclofenac sodium [11]. Anandachockalingam et al. obtained similar results when they used green-synthesized Ag-NPs to significantly limit albumin denaturation [8]. These investigations validate plant-mediated nanoparticle systems as anti-inflammatory drugs and confirm the current findings. Our study is also similar to the study by Nedumaran et al., who studied anti-inflammatory activity in a green-synthesized ZnO-NP study using *Echinacea* extract to produce ZnO-NPs and assessed anti-inflammatory activity via membrane stabilization and BSA assays. They observed concentration-dependent anti-inflammatory effects, supporting the utility of plant-derived ZnO-NPs in reducing inflammation [12].

The CaO-NP formulation’s anti-inflammatory properties were further supported by the MSA. Stabilization of erythrocyte membranes is considered analogous to stabilization of lysosomal membranes, thereby preventing the release of inflammatory mediators and hydrolytic enzymes. The high percentage of membrane stabilization observed in this study indicates the ability of the synthesized nanoparticles to protect cellular damage against inflammatory damage. Plant-derived nanoparticles, such as CuO-NPs and Ag-NPs, which showed notable stability in vitro, have been shown to have similar membrane-protective effects [13]. This kind of membrane maintenance is especially important for inflammatory diseases that impact epithelial tissues, such as ulcerative lesions and oral mucositis. This is consistent with a study by Chellathurai et al., who observed that copper and silver nanoparticles exhibited increasing anti-inflammatory activity (suppression of protein denaturation and membrane stabilization) with increasing concentration, reinforcing the broader role of plant-derived metal oxide nanoparticles in inflammation mitigation. A previously reported review on green synthesis of metal oxide nanoparticles highlighted the biomedical relevance and their application as an anti-inflammatory drug [5].

The observed biological activity may be attributed to the synergistic interaction between phytochemical components of *S. cumini* and the CaO-NPs. The fruit of *S. cumini* is rich in anthocyanins, flavonoids, and polyphenols that are known to have anti-inflammatory and antioxidant qualities [14]. These phytochemicals stay attached to the surface of the nanoparticles and function as capping and reducing agents during green synthesis, which may increase biological activity. Phytochemical capping can enhance anti-inflammatory and antioxidant responses by modifying oxidative stress pathways and protein interactions, according to reviews on green-synthesized metal oxide nanoparticles [2]. The bioactive phytochemicals present on the nanoparticle surface may also stabilize proteins, protect biological membranes from lysis, and reduce oxidative stress, thereby attenuating inflammatory responses. Similar mechanisms have been proposed for other green-synthesized metal oxide nanoparticles, including Ag-NPs, ZnO-NPs, and CuO-NPs, in previous studies [1,5,9].

CaO-NPs also offer a large surface area and increased biomolecule interaction, which may help stabilize proteins and membranes. According to Munusamy and Shanmugam, when produced from plant extracts, green-synthesized CaO-NPs show encouraging biological activity and surface functionalization [15]. In a similar study, Kazi et al. found that biosynthesized CaO-NPs had acceptable cytocompatibility and advantageous biological interactions in dental applications, indicating their translational potential in oral therapeutic systems [2]. The anti-inflammatory activity of *S. cumini*-mediated CaO-NPs may be attributed to the combined effects of phytochemical capping and enhanced nanoparticle surface reactivity.

The application of plant-based nanoformulations for oral inflammatory disorders is further supported by recent nanomedicine research. Significant anti-inflammatory effects were shown in vitro by comparative studies of nanocomposite herbal mouth rinses [12]. Furthermore, in experimental models, *S. cumini*-based nanofibrous systems have demonstrated improved wound healing and anti-inflammatory activity [4]. Collectively, these findings support the growing evidence that green-synthesized metal oxide nanoparticles represent promising candidates for the development of novel anti-inflammatory therapeutics, particularly in oral healthcare applications.

It is crucial to understand that protein denaturation and membrane stabilization experiments are still preliminary screening models, despite the encouraging in vitro results. Confirming molecular pathways and safety profiles requires thorough investigation in cell-based systems, including cytokine profiling (e.g., tumor necrosis factor-alpha, interleukin (IL)-6, IL-1β) and in vivo models of oral inflammation. Furthermore, as recent reviews of green-synthesized metal oxide nanoparticles have shown, comprehensive toxicity and stability evaluations are crucial before clinical translation [2].

Study limitations

This study has a few limitations. First, the anti-inflammatory activity was evaluated exclusively using in vitro assays, which may not fully replicate the complex inflammatory response occurring in living organisms. Second, molecular mechanisms responsible for the observed anti-inflammatory effects were not investigated. Finally, cytotoxicity, biocompatibility, and safety assessment were not performed. Future studies should focus on mechanistic investigations and toxicity evaluation to establish the therapeutic potential. The novelty of the present study lies in the green synthesis of CaO-NPs using *S. cumini* fruit extract and the direct comparative evaluation of the in vitro anti-inflammatory activity of the fruit extract and its corresponding nanoparticles using multiple mechanistic assays, including the EA denaturation assay, BSA denaturation assay, and MSA. Unlike previous studies that primarily focused on the antimicrobial or antioxidant properties of plant-mediated CaO-NPs, the present study provides preliminary evidence of their anti-inflammatory potential, thereby expanding their possible biomedical applications.

## Conclusions

Within the limitations of this in vitro study, green-synthesized CaO-NPs mediated by *S. cumini* fruit extract demonstrated concentration-dependent anti-inflammatory activity in protein denaturation and membrane stabilization assays. These findings provide preliminary evidence supporting the anti-inflammatory potential of plant-mediated CaO-NPs. However, further mechanistic investigations, comprehensive statistical validation, and in vivo studies are required to confirm their biological efficacy, safety, and potential applicability in the management of inflammatory conditions.
